# Heterogeneous ribonuclear protein A3 (hnRNP A3) is present in dipeptide repeat protein containing inclusions in Frontotemporal Lobar Degeneration and Motor Neurone disease associated with expansions in *C9orf72* gene

**DOI:** 10.1186/s40478-017-0437-5

**Published:** 2017-04-21

**Authors:** Yvonne S. Davidson, Louis Flood, Andrew C. Robinson, Yoshihiro Nihei, Kohji Mori, Sara Rollinson, Anna Richardson, Bridget C. Benson, Matthew Jones, Julie S. Snowden, Stuart Pickering-Brown, Christian Haass, Tammaryn Lashley, David M. A. Mann

**Affiliations:** 1 Division of Neuroscience and Experimental Psychology, School of Biological Sciences, Faculty of Biology, Medicine and Health, University of Manchester, Salford Royal Hospital, Salford, M6 8HD UK; 2 Biomedical Centre (BMC), Biochemistry, Ludwig-Maximilians Universitat Munchen, Munich, Germany; 3 Department of Psychiatry, Osaka University Graduate School of Medicine, Osaka, Japan; 4 Division of Neuroscience and Experimental Psychology, School of Biological Sciences, Faculty of Biology, Medicine and Health, University of Manchester, A V Hill Building, Manchester, M13 9PT UK; 5 Cerebral Function Unit, Greater Manchester Neurosciences Centre, Salford Royal Hospital, Stott Lane, Salford, M6 8HD UK; 6 Department of Molecular Neuroscience, University College London, Institute of Neurology, Queen Square Brain Bank for Neurological Disorders, 1 Wakefield St, London, WC1N 1PJ UK; 7 German Centre for Neurodegenerative Diseases (DZNE) Munich, Munich, Germany; 8 Munich Cluster for Systems Neurology (SyNergy), Munich, Germany

**Keywords:** Frontotemporal Lobar Degeneration, Motor neuron disease, Heterogeneous ribonuclear proteins, *C9orf72* gene, Dipeptide repeat proteins

## Abstract

Frontotemporal Lobar Degeneration (FTLD) encompasses certain related neurodegenerative disorders which alter behaviour, personality and language. Heterogeneous ribonuclear proteins (hnRNPs) maintain RNA metabolism and changes in their function may underpin the pathogenesis of FTLD. Immunostaining for hnRNP A1, A2/B1 and A3 was performed on sections of temporal cortex with hippocampus from 61 patients with FTLD, stratified by pathological hallmarks into FTLD-tau and FTLD-TDP type A, B and C subtypes, and by genetics into patients with *C9orf72* expansions, *MAPT* or *GRN* mutations, or those without known mutation. Four patients with Motor Neurone Disease (MND) with *C9orf72* expansions and 10 healthy controls were also studied. Semi-quantitative analysis assessed hnRNP staining intensity in dentate gyrus (DG) and CA4 region of hippocampus, and temporal cortex (Tcx) in the different pathological and genetic groups.

Immunostaining for hnRNP A1, A2/B1 and A3 revealed no consistent changes in pattern or amount of physiological staining across any of the pathological or genetic groups. No immunostaining of any inclusions resembling TDP-43 immunoreactive neuronal cytoplasmic inclusions or dystrophic neurites, was seen in either Tcx or DG of the hippocampus in any of the FTLD cases investigated for hnRNP A1, A2/B1 and A3. However, immunostaining for hnRNP A3 showed that inclusion bodies, resembling those TDP-43 negative, p62-immunopositive structures containing dipeptide repeat proteins (DPR) were variably observed in hippocampus and cerebellum. The proportion of cases showing hnRNP A3-immunoreactive DPR, and the number of hnRNP A3-positive inclusions within cases, was significantly greater in DG than in cells of CA4 region and cerebellum, but the latter was significantly less in all three regions compared to that detected by p62 immunostaining.

**Electronic supplementary material:**

The online version of this article (doi:10.1186/s40478-017-0437-5) contains supplementary material, which is available to authorized users.

## Introduction

Frontotemporal Lobar Degeneration (FTLD) is a clinically, pathologically and genetically heterogeneous disorder affecting principally the frontal and temporal lobes of the brain. After Alzheimer’s disease, it is the second most common neurodegenerative disorder to affect people before the age of 65 years. Three major syndromes are recognised clinically [32]. One syndrome is characterised by changes in behaviour and personality, accounting for around 75% of all cases of FTLD, is known as behavioural variant frontotemporal dementia (bvFTD), whereas the other two syndromes are disorders of language. Semantic dementia (SD) (also known as semantic variant of primary progressive aphasia or svPPA) is a disorder of naming and word finding, whereas Progressive Non-Fluent Aphasia (PNFA) (also known as nfvPPA) is represented by an inability to construct language such that speech becomes hesitant and stuttering, becoming grammatically and contextually incorrect [32]. The amyotrophic lateral sclerosis (ALS) form of Motor Neurone Disease (MND) is seen in about 15% of patients with bvFTD, giving FTD-MND (FTD-ALS), but is only rarely combined with either SD or PNFA [32].

Three different pathologies can be present, all characterised by abnormal neuronal, and sometimes glial, accumulations of aggregated proteins. In about 45% cases, neuronal intracytoplasmic inclusions (NCI) composed of the microtubule associated protein, tau are seen as neurofibrillary tangle-like structures (NFT) or more amorphous tau deposits (pre-tangles) or more rounded, tau-immunoreactive inclusions, known as Pick bodies [31]. Such cases are termed FTLD-tau [20]. In about 50% of remaining cases [31], the RNA and DNA binding protein, TDP-43, is present within NCI, neuritic processes (dystrophic neurites, DN) or neuronal intranuclear inclusions (NII) [1, 26]; such cases are collectively termed FTLD-TDP [20]. The proportion of each of these histological features varies according to clinical phenotype or genetic background, and these have been used to provide a neuropathological classification of FTLD-TDP subtypes [20]. FTLD-TDP subtype A is applied when NCI and DN are both commonly present, type B when NCI numerically predominate over DN, type C when DN predominate over NCI and type D when NII are most common type of histological change. Most of the remaining 5% cases show NCI composed of the protein, Fused in Sarcoma (FUS) (also known as Translocation in Liposarcoma, TLS), and known as FTLD-FUS [20].

Both TDP-43 and FUS belong to the heterogeneous nuclear riboprotein (hnRNP) family of which there are more than 20 members, labelled A1-U, ranging in size from 34 to 120KDa [6, 28]. These are RNA- and DNA-binding proteins, and serve as RNA-splicing and transcription regulators, shuttling between nucleus and cytoplasm, thereby controlling cellular levels of protein synthesis [6, 28]. In the nucleus, TDP-43 binding encourages RNA stability, whereas in the cytoplasm it associates with stress granules and non-coding RNAs for post-transcriptional metabolism of RNA and transport. In FTLD-TDP there is a ‘clearing’ of normal physiological TDP-43 from the nucleus with its accumulation within the cytoplasmic as NCI, DN or NII [1, 26]. However, the precise mechanism(s) directing this pathological change remain unclear.

Other members of hnRNP family may also play a role in the pathogenesis of FTLD, especially in patients with expansions in *C9orf72*. In pull down assays, Mori et al. reported hnRNP A3 to bind specifically to the G4C2 repeats, and to colocalize with dipeptide repeat protein (DPR) inclusions by immunohistochemistry in a proportion of hippocampal dentate gyrus (DG) and CA4 neurons, and cerebellar granule cells [22]. In this same study, hnRNPs A1, A2/B1 and K were also shown to bind to the G4C2 sequence, though these did not co-localize with NCI [22]. In a follow up study, Mori and colleagues showed that knock-down of nuclear hnRNP A3 lead to an increase in poly-GA, poly-GR and poly-GP protein production with subsequent aggregation into DPR inclusion bodies, nuclear RNA foci and mislocalisation of TDP-43 [23].

Elsewhere, Cooper-Knock et al. [8] reported co-localisation of hnRNP A1 and hnRNP H/F with sense RNA foci in DG granule cells in patients with expansions in *C9orf72*. In a follow-up study, the same group demonstrated a similar co-localisation with antisense foci in cerebellar Purkinje cells [7]. DPR might therefore trap hnRNPs thereby inhibiting their function. Furthermore, mutations in hnRNP A1 and A2/B1 have been reported to cause multisystem proteinopathy and amyotrophic lateral sclerosis [14], but not all groups have detected such mutations in FTLD or MND [5, 15, 30].

In the present study, we have investigated patterns of hnRNP A1, A2/B1 and A3 immunostaining in patients with FTLD, specifically in respect of the genetic mutations and histological variants present, in order to gain insight into understanding the role of these proteins in driving the pathogenetic cascade. We find that a proportion of DPR within the granule cells of the DG of the hippocampus and cerebellum also contain hnRNP A3 protein in patients bearing expansions in *C9orf72*, and might therefore contribute to the pathogenetic cascade in this particular subgroup of FTLD patients.

## Materials and methods

### Patients

The study consisted of two sets of cases comprising 75 subjects in total. One group of 59 patients had been recruited through Manchester Brain Bank (MBB), 55 with a clinical diagnosis of FTLD (31 males, 24 females; cases #1-11 and 16–59), four with a diagnosis of Motor Neurone Disease (MND) (4 males; cases #12-15) together with 10 healthy control subjects (three males, seven females; cases #55-64) (see Additional file 1: Table S1). The brains of these patients had been consecutively acquired by MBB over the years 1986 to present. All patients were from the North West of England and North Wales, and tissues were obtained through appropriate consenting procedures for the collection and use of the human brain tissues. The other group of 6 patients with a clinical diagnosis of FTLD (two males, four females; cases #70-75) (see Additional file 1: Table S1) was recruited through Queens Square Brain Bank (QSBB). The brains of these six patients had been acquired by QSBB over the years 2010 to present. All patients were from London and South of England, and tissues were again obtained through appropriate consenting procedures for the collection and use of the human brain tissues. All 61 FTLD patients fulfilled relevant clinical diagnostic criteria [12, 24, 29], 55 of which having been investigated longitudinally within specialist dementia clinics at Salford Royal Hospital using the Manchester Neuropsychological Profile (Man-NP) [33, 34] to determine and characterise the nature of their dementia. The other six patients (cases #70-75) had been investigated at Dementia research Centre, Queen Square, London. The four patients with MND fulfilled El Escorial criteria [4].

Of the combined 61 FTLD patients, 30 had been clinically diagnosed with bvFTD (14 males, 16 females; cases #1-6,18,19,22-25,28-30,49-59,70,73-75), 13 with bvFTD + MND (nine males, four females; cases #7-11,31-38), 8 with PNFA (four males, four females; cases #16,17,20,21,26,27,71,72) and 10 with SD (six males, four females; cases #39-48) (Additional file 1: Table S1). Histologically, the FTLD group comprised 25 patients with FTLD-TDP type A (cases #1-5,16-30, 70–74), 15 with FTLD-TDP type B (cases #6-11,31-38,75), 10 with FTLD-TDP type C (cases #39-48) and 11 with FTLD-tau (cases #49-59). Furthermore, within the FTLD group there were 17 patients with expansions in *C9orf72* (cases #1-10,70-75), nine with *GRN* mutations (cases #16-24), 10 patients with intronic mutations in *MAPT* (cases #49-59) and 24 without known mutation (cases #25-48). All four patients with MND (cases# 12–15) bore expansion in *C9orf72*.

Comparison of the four FTLD pathology, and MND, patient groups showed significant differences in mean age at onset of disease (F_4,60_ = 4.9, *p* = 0.002), mean age at death (F_4,60_ = 9.1, *p* < 0.001) and duration of illness did differ (F_4,60_ = 7.5, *p* < 0.001). Patients with FTLD-tau had earlier age at onset than those with FTLD-TDP type A and FTLD-TDP type C subgroups (*p* = 0.001 and 0.034, respectively), but not compared to FTLD-TDP type B or MND subgroups. None of the FTLD-TDP groups differed from each other, or from MND group, in this respect. Patients with FTLD-tau also had earlier age at death than those with FTLD-TDP type A and FTLD-TDP type C subgroups (*p* = 0.01 and 0.002, respectively), but not compared to FTLD-TDP type B or MND subgroups. Patients with FTLD-TDP type B, and those with MND, had earlier age at death than those with FTLD-TDP-type A (*p* = 0.013 and *p* = 0.003, respectively) and FTLD-type C (*p* = 0.001 and *p* = 0.002, respectively). Consequently, because of their MND, patients with FTLD-TDP type B, and those with MND, had a shorter duration of illness than those with FTLD-type C (*p* = 0.001 in both instances) and FTLD-tau (*p* = 0.017 and *p* = 0.016 respectively) (Table 1). The healthy control group was also significantly older at death (*p* < 0.001) than each of the FTLD subgroups (Table 1).

**Table 1:** Selected clinical, neuropathological and genetic details on patients studied. FTLD = Frontotemporal Lobar degeneration; MND = Motor Neurone Disease

Group	M/F	Age at onset (y)	Age at death (y)	Duration of illness (y)
FTLD-TDP type A (*n* = 25)	14/11	61.0 ± 5.9	69.0 ± 5.1	8.0 ± 3.3
FTLD-TDP type B (*n* = 15)	9/6	57.1 ± 7.4	62.3 ± 8.0	5.3 ± 4.5
FTLD-TDP type C (*n* = 10)	6/4	59.9 ± 7.1	71.8 ± 5.7	11.9 ± 5.0
FTLD-tau (*n* = 11)	4/7	51.4 ± 6.4	61.4 ± 5.4	10.0 ± 3.1
MND (*n* = 4)	4/0	53.3 ± 7.3	56.3 ± 8.3	3.0 ± 1.4
FTLD/MND *C9orf72* expansion (*n* = 21)	13/8	57.3 ± 6.0	63.5 ± 6.4	6.2 ± 4.1
FTLD *GRN* mutation (*n* = 9)	5/4	60.7 ± 5.6	69.3 ± 4.1	8.7 ± 3.9
FTLD No mutation (*n* = 24)	15/9	60.2 ± 7.8	68.5 ± 8.9	8.4 ± 4.9
FTLD *MAPT* mutation (*n* = 11)	4/7	51.4 ± 6.4	61.4 ± 5.4	10.0 ± 3.1
Healthy Controls (*n* = 10)	3/7	na	83.3 ± 7.6	na

Comparison of the four FTLD genetic patient groups also showed significant differences in mean age at onset of disease (F_3,61_ = 4.9, *p* = 0.004) and mean age at death (F_3,61_ = 4.1, *p* = 0.010) though duration of illness did not differ significantly (F_3,61_ = 2.2, *p* = 0.095). Patients with *MAPT* mutation had earlier age at onset than those with *GRN* mutation (*p* = 0.017) and those without known mutation (*p* = 0.004), but not those with *C9orf72* expansion (*p* = 0.092) which in turn did not differ from *GRN* and no mutation groups. Mean age at death was significantly earlier in *MAPT* than the no mutation group (*p* = 0.034) but otherwise there were no significant differences between all other group pairings (Table 1).

### Histological methods

Paraffin sections were cut at 6μm from formalin fixed blocks of temporal lobe (BA21/22) (to include the posterior hippocampus) from all 61 FTLD cases, four MND cases and the 10 healthy control cases, and from cerebellar cortex (to include dentate nucleus) of all 21 carriers of an expansion in *C9orf72*. Following titration (dilutions 1:100 to 1:2000) to determine optimal immunostaining, antibodies were identically employed in a standard IHC protocol, as described previously [9, 21]. The following antibodies were employed: hnRNP A1 (Santa Cruz, sc374526, 1:2000), hnRNP A2/B1 (Santa Cruz, sc374052, 1:500), hnRNP A3 (Santa Cruz, sc133665, 1:100 and Sigma AV41195, 1:150), TDP-43 (10782-2-AP antibody, Proteintech, Manchester, UK, 1:1000), tau (AT8, Innogenetics, Antwerp, Belgium, 1:750) and p62 (p62-lck ligand, rabbit polyclonal antibody, B D Biosciences, Oxford, UK, 1:100) proteins. For each antibody, antigen unmasking was performed by pressure cooking in citrate buffer (pH 6.0, 10mM) over a 30 min period to include warming and cooling times, reaching 123° Celsius for 30 s, and >15 psi pressure. Sections of temporal cortex and hippocampus from 54/55 FTLD and all 10 control cases obtained from MBB were immunostained using TDP-43, tau, hnRNP A1, A2 and A3 (Santa Cruz) antibodies. Sections from six QSBB cases were immunostained using TDP-43 and hnRNP A3 (Santa Cruz). Sections of hippocampus and cerebellum from all 21 carriers of an expansion in *C9orf72* from both MBB and QSBB were additionally immunostained for p62 protein and hnRNP A3 (Sigma antibody).

### Pathological assessment

Sections were examined microscopically for the appearance of intracellular distribution of immunostaining within neurons of the temporal cortex (Tcx), DG and CA4 region of the hippocampus and for the presence of any hnRNP immunostained structures (NCI) resembling those seen in TDP-43 or tau immunostaining. These regions were chosen because Tcx and DG of the hippocampus are involved with TDP-43 pathology in all forms of FTLD-TDP, or tau pathology in those patients with *MAPT* mutation. Moreover, the CA4 region of the hippocampus and cerebellar cortex were included because these are among the principal regions affected by DPR pathology in patients with expansions in *C9orf72* [4, 21]. The magnitude of physiological neuronal hnRNP staining in each region was scored semi-quantitatively at an objective magnification of x25 (overall magnification of x250), employing the following rating scale:

- 0 = No staining present.
- 0.5 = rare (ie 1–5) cells per section showing weak nuclear and/or cytoplasmic staining.
- 1 = 1–5 cells showing weak nuclear and/or cytoplasmic staining per x250 microscope field.
- 2 = 5–10 cells showing moderate nuclear and/or cytoplasmic staining per x250 microscope field.
- 3 = more than 10 cells showing strong nuclear and/or cytoplasmic staining per x250 microscope field.

The magnitude of intraneuronal hnRNP A3 and p62 inclusion body immunostaining in areas CA4 and DG of the hippocampus, and granule cells of the cerebellum, was scored semi-quantitatively at an objective magnification of x25 (overall magnification of x250), employing the following rating scale:

- 0 = No inclusions present.
- 0.5 = rare (ie 1–5 inclusions per section).
- 1 = few (ie 1–5 inclusions per x250 microscope field).
- 2 = moderate (ie 5–10 inclusions per x250 microscope field).
- 3 = many (ie 10–50 inclusions per x250 microscope field).
- 4 = very many (ie more than 50 inclusions per x250 microscope field).

Scoring of staining for all cases and immunostains, as presented here, was performed by a single experienced observer (DMAM) blinded to clinical, histopathological and genetic status. However, a subset of 20 hnRNP A2 immunostained cases, selected at random, to cover all pathological subgroups, was chosen for scoring by a second, less experienced, observer (AR). Independent scoring of cases was performed by both observers and showed good agreement between all pairs (κ = 0.6, *p* = 0.000), with 70% of scores being the same, and no score between cases differing by more than one grade in any pairwise comparison.

### Statistical analysis

Rating data was entered into an excel spreadsheet and analyzed using Statistical Package for Social Sciences (SPSS) software (version 17.0). The FTLD patients were stratified according to genetic and pathological subtype for statistical analysis of the effect of each mutation and underlying pathology on the pattern of the staining for each hnRNP antibody. Comparisons of semi-quantitative scores for intensity of physiological A1 and A2/B1 hnRNP immunostaining in Tcx, DG and CA4 region of the hippocampus, and hnRNP A3 (Sigma) and p62 inclusion body staining in DG and CA4 region of the hippocampus and cerebellar cortex, were all performed using Kruskal-Wallis test with post-hoc Mann-Whitney test where Kruskal-Wallis yielded a significant difference between antibody staining scores. Comparison of scores for hnRNP A3 (Sigma) and p62 inclusion body staining between DG and CA4 region of the hippocampus and cerebellar cortex were made using Wilcoxon matched pairs test. Group comparisons of age at onset, age at death and duration of illness were made using ANOVA with post hoc Tukey test. In all instances, significance levels were set at *p* < 0.05.

## Results

### TDP-43 immunostaining

TDP-43- and tau-immunostaining was employed to classify the 61 FTLD patients into their respective histological subgroups (FTLD-TDP subtypes A, B or C and FTLD-tau) according to the form and distribution of the TDP-43 or tau-immunoreactive inclusions (NCI, DN) present (see Mackenzie et al. 2011 [20]). Consequently, as would be expected when FTLD-tau and control cases were included, the overall degree of TDP-43 immunostaining (irrespective of histological type) differed between the five pathological groups in both the cerebral cortex (F_4,50_ = 47.4, *p* < 0.001) and dentate gyrus (F_4,50_ = 48.2, *p* < 0.001) with all three FTLD-TDP subtypes differing significantly from FTLD-tau and control groups (*p* < 0.001 in every instance). However, there were no significant differences in the overall degree of TDP-43 immunostaining between FTLD-TDP type A, type B and type C groups.

### hnRNP A1

**Fig. 1:**
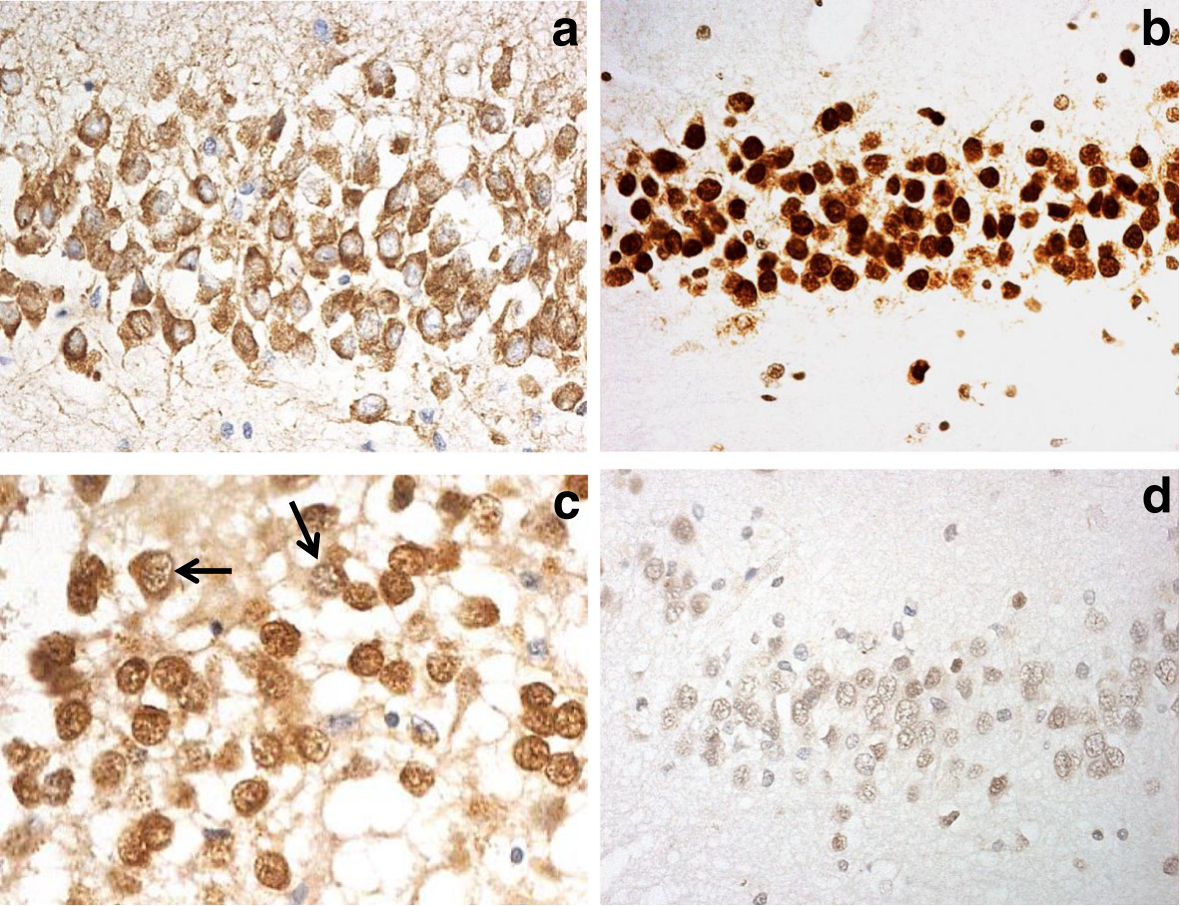
Immunostaining for hnRNPs in dentate gyrus of the hippocampus in normal human brain. hnRNP A1 is localised mostly in cell cytoplasm (**a**), whereas hnRNP A2 (**b**,**c**) and hnRNP A3 (**d**) are mostly nuclear. In cases of FTLD-TDP type C there is some ‘nuclear clearing’ of hnRNP A2 (*arrowed* in **c**). Immunoperoxidase, microscope magnification, ×400

All FTLD (*n* = 54) and control (*n* = 10) cases studied, showed some degree of immunostaining for hnRNP A1, ranging from weak, through to strong, in intensity. Immunostaining in FTLD cases was usually nuclear and cytoplasmic, rather than nuclear alone, though sometimes cytoplasm was predominantly immunostained (Fig. 1a). In control cases some cytoplasmic staining was seen, as well as the expected nuclear staining, but this was generally weaker than observed in FTLD cases. In the FTLD cases, there did not appear to be any obvious group differences with respect to either the pattern of staining across the three regions, or the range of staining intensity achieved, between any of the pathological, or genetic, groups.

However, semi-quantitative analysis revealed no significant difference in scores for hnRNP A1 immunostaining between any of the five pathological groups for DG (*χ* ^2^ = 1.5, *p* = 0.833) and CA4 (*χ* ^2^ = 3.9, *p* = 0.421) regions of hippocampus, though there was a trend towards significance in Tcx (*χ* ^2^ = 9.7, *p* = 0.047), driven by significantly lower scores in FTLD-TDP type C group compared to FTLD-TDP type B (*p* = 0.01), FTLD-tau (*p* = 0.013) and control (*p* = 0.015) groups. Similarly, comparing the four genetic groups, there was a significant difference in scores for CA4 (*χ* ^2^ = 11.2, *p* = 0.025) region of hippocampus, but not for DG (*χ* ^2^ = 5.1, *p* = 0.274) and Tcx (*χ* ^2^ = 8.3, *p* = 0.081), driven in CA4 region by there being significantly less staining in the non-genetic group compared to *C9orf72* (*p* = 0.003), *GRN* (*p* = 0.031), and *MAPT* (*p* = 0.029) groups.

No structures resembling TDP-43 immunoreactive NCI or DN, in either Tcx or DG, or DPR immunoreactive NCI in CA4 neurons, was seen on immunostaining for hnRNP A1.

### hnRNP A2/B1

Again, all FTLD (*n* = 54) and all control (*n* = 10) cases studied, showed some degree of immunostaining for hnRNP A2/B1. This was usually strongly nuclear (Fig. 1b), though on occasions within the FTLD cases both nucleus and cytoplasm were immunostained. There did not appear to be any obvious group differences with respect to either the pattern of staining across the three regions, or the range of staining intensity achieved, between any of the pathological, or genetic, groups. However, it was noted that some cases of FTLD-TDP type C showed a clearing of nuclear staining from some of the cells within DG (Fig. 1c).

Semi-quantitative analysis also revealed no significant difference in scores for hnRNP A2/B1 immunostaining between any of the five pathological (Tcx, *χ* ^2^ = 9.1, *p* = 0.058; DG, *χ* ^2^ = 1.5, *p* = 0.833; CA4, *χ* ^2^ = 3.9, *p* = 0.421) or four genetic (Tcx, *χ* ^2^ = 6.4, *p* = 0.171; DG, *χ* ^2^ = 2.3, *p* = 0.672; CA4, *χ* ^2^ = 3.0, *p* = 0.553) groups.

Again, no structures resembling TDP-43 immunoreactive NCI or DN, in either TCX or DG, or DPR immunoreactive NCI in CA4 neurons, was seen on immunostaining for hnRNP A/B1.

### hnRNP A3

No immunostaining was obtained in any case, FTLD or control, when using the Santa Cruz hnRNP A3 antibody, at all antibody dilutions tested (1:50–1:500). However, when Sigma hnRNP A3 antibody was used on a selection of the FTLD and control cases, which included all cases bearing expansions in *C9orf72* and representative cases from the other FTLD groups, some degree of immunostaining for hnRNP A3 was seen in most instances. This was usually nuclear (Fig. 1d), ranging from very weak to moderately strong, though on rare occasions within FTLD cases both nucleus and cytoplasm was immunostained. There did not appear to be any obvious group differences with respect to either the pattern of nuclear or cytoplasmic staining across Tcx and hippocampus, or the range of staining intensity achieved, between any of the pathological or genetic groups. No semi-quantitative comparisons between pathological or genetic groups were performed, as the number of cases examined overall, particularly those not bearing expansion in *C9orf72*, was insufficient for meaningful statistical analysis.

**Fig. 2:**
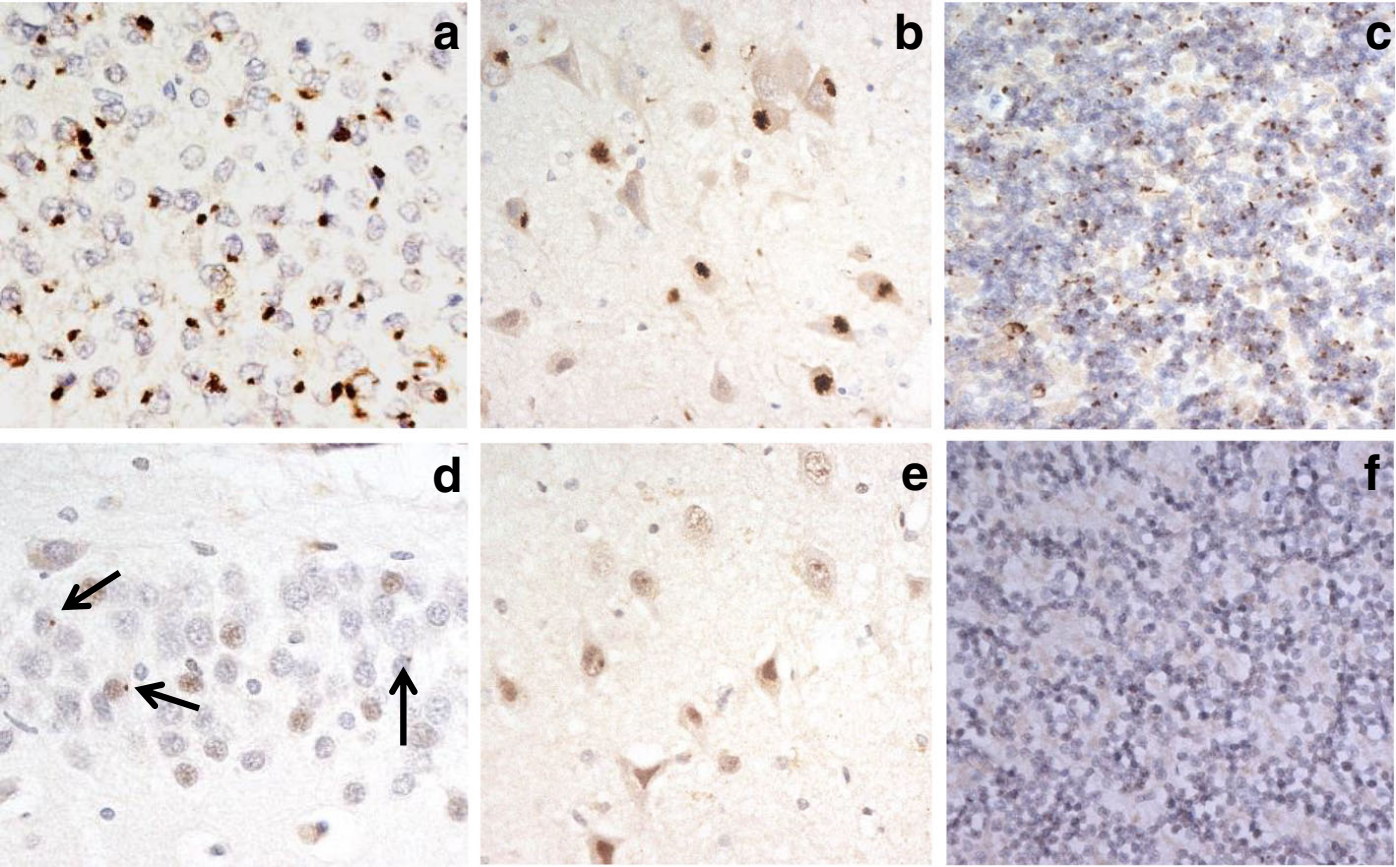
Immunostaining for p62 (**a**-**c**) and hnRNP A3 (**d**-**f**) in dentate gyrus (**a**,**d**) and CA4 region (**b**,**e**) of hippocampus, and in cerebellum (**c**,**f**), in cases of FTLD-TDP associated with expansions in *C9orf72* gene. There are abundant p62-immunoreactive neuronal cytoplasmic inclusions in dentate gyrus (**a**) and CA4 region (**b**) of hippocampus, and in cerebellum (**c**), though only a small proportion of cells in dentate gyrus show similar appearing hnRNP A3-immunoreactive inclusions (*arrowed* in **d**), but none are present in CA4 region (**e**) or cerebellum (**f**). Immunoperoxidase, microscope magnification, ×400

No immunostaining of any inclusions resembling TDP-43 immunoreactive NCI or DN, was seen in either Tcx or DG of the hippocampus in any of the FTLD cases investigated. However, immunostaining for hnRNP A3 showed that inclusion bodies, resembling those p62-immunoreactive structures (NCI) containing DPR (Fig. 2a-c), were variably present in the hippocampus and cerebellum (Fig. 2d-f). NCI were most often, but numerically only sparsely, observed in granule cells of the DG in 17/21 cases bearing expansions in *C9orf72* (Fig. 2d), and were only rarely seen in cells of CA4 region of the hippocampus in 1/21 cases (Fig. 2e), and not at all in granule cells of the cerebellum in 12/21 cases (Fig. 2f), though very rarely isolated NCI were seen in the other nine cases. Hence, the proportion of cases showing DPR in granule cells of DG was significantly greater than that for cells of CA4 region (*χ* ^2^ = 24.9; *p* = <0.001) or cerebellum (*χ* ^2^ = 8.4; *p* = <0.003), with the proportion of cases showing DPR in cerebellum being greater than that of CA4 neurons (*χ* ^2^ = 6.5; *p* = <0.01).

While the overall scores for the numerical severity of p62 positive inclusions (DPR) did not significantly differ between granule cells of DG, CA4 region and cerebellum (*χ* ^2^ = 2.95, *p* = 0.228), the scores for those which were A3-immunoreactive did differ significantly (*χ* ^2^ = 31.2, *p* = 0.000) with the scores for A3-positive inclusions in cells of DG being greater than those in CA4 region (*p* = 0.000) and cerebellum (*p* = 0.000), and those for the cerebellum being greater than those for CA4 region (*p* = 0.000). The number of inclusions seen on hnRNP A3 immunostaining was clearly much less than that seen on p62 immunostaining across all three regions (Fig. 2). Accordingly, semi-quantitative analysis revealed significantly lower scores for hnRNP A3 inclusion body staining, compared to that of p62 immunostaining, for DG granule cells in hippocampus (*p* = 0.000) and cerebellum (*p* = 0.000) and for CA4 neurones of the hippocampus (*p* = 0.000).

## Discussion

In the present study, we have investigated the pattern of hnRNP A1, A2/B1 and A3 immunostaining across a range of clinical, pathological and genetic forms of FTLD and MND. Microscopically, there appeared to be increased cytoplasmic staining for hnRNP A1, and to a lesser extent hnRNP A2/B1, across each of the FTLD pathological or genetic groups. While this might reflect an increased physiological expression of these hnRNPs, it could also represent a cellular re-localisation of protein from nucleus to cytoplasm. However, given the wide range of semi-quantitative scores for hnRNP A1 and A2/B1 across each of the pathological groups, the microscopic observations could not be substantiated by semi-quantitative statistical analysis. Nonetheless, Gami-Patel and colleagues also noted an increased cytoplasmic staining of hnRNP A1 in cases with FTLD-FUS [11]. Together, these data suggest there might be a derangement of movement of hnRNP A1, and other hnRNP proteins, across all pathological forms of FTLD beyond that involving just TDP-43 or FUS.

No immunoreactive structures, resembling those seen in FTLD cases on tau or TDP-43 immunostaining, were seen following immunostaining for hnRNP A1, A2/B1 or A3, consistent with previous findings [11]. Such observations would be consistent with genetic studies showing that mutations in hnRNP A1 and hnRNP A2/B1 genes, which might be anticipated to result in molecular or pathological changes, are extremely rare events in both FTLD and MND [5, 14, 15, 30]. Interestingly, on the other hand, a proportion of FUS-positive inclusions in FTLD-FUS, especially cases of the Neuronal Intermediate Filament Inclusion Body Disease form of FTLD-FUS, have been reported to contain hnRNP A1 protein, along with other hnRNPs to a lesser extent [25]. This finding would be consistent with studies showing disruption of FET proteins, transportin-1 (TRN1), TAF15 and EWS in FTLD-FUS [3, 10], given that hnRNP A1 can act as a cargo protein for TRN1 in TRN1-mediated nuclear import [16].

However, when using Sigma hnRNP A3 antibody, NCI resembling those seen with p62 or DPR immunostaining were variably seen in granule cells of DG of the hippocampus in 17/21 FTLD cases with *C9orf72* expansion, but only rarely so in a single case in neurons of CA4 region. hnRNP A3 immunoreactive inclusions were also very rarely seen in cerebellum in 9/21 cases. Semi-quantitative comparisons showed that the number of hnRNP A3 immunoreactive inclusions was significantly less than those seen on p62 immunohistochemistry of the same cases in all three regions.

To our knowledge there has only been three previous studies investigating the role of hnRNP A3 in FTLD [22, 23]. In 2013, Mori and colleagues reported hnRNP A3 to bind specifically to G4C2 repeats in pull down assays, and to colocalize by immunohistochemistry with p62/DPR NCI in a proportion of hippocampal DG and CA4 neurons, and cerebellar granule cells [22]. As we have found here, Mori et al. noted that the number of A3 immunoreactive NCI was significantly less than those detected by p62 immunostaining in these same brain regions, with DG granule cells again showing the greatest number of NCI and CA4 neurons the least [22]. Although hnRNPs A1, A2/B1 and K were also shown to bind to the G4C2 sequence in pull down assays, these did not co-localize with NCI by immunohistochemistry [22]. However, in this particular study there were some patients with FTLD-TDP, but without *C9orf72* expansion, who also showed rare hnRNP A3-containing neurites, but not inclusion bodies, suggesting that hnRNP A3 dysfunction, as well as other hnRNPs, could play a wider role in mRNA deregulation and the pathophysiology of FTLD. In the later study [23], hnRNP A3 was found to co-localise with a proportion of poly-GA contain DPR in hippocampal DG granule cells. Moreover, poly-GA DPR were over 50% higher in cases with low nuclear hnRNP A3 expression compared to cases with high expression [23]. Recently, Boeynaems et al. also observed hnRNP A3 immunostaining of a few DPR in DG granule cells in six patients with *C9orf72* associated FTLD [2].

The present study is consistent with these previous findings [2, 22, 23] as regards both the pattern of immunostaining for hnRNP A1 and A2/B1, and the presence of hnRNP A3 immunoreactivity in a subset of hippocampal and cerebellar DPR containing NCI and its relationship to p62 and DPR (poly-GA) immunostaining. However, in contrast to Mori et al. [22], we did not find hnRNP A3 immunostaining of other structures such as NCI, DN or NII in any other FTLD cases despite intensive survey.

hnRNP A3 is a member of the hnRNP A/B family of RNA-binding proteins that, in common with other RNA-binding proteins, contains two N-terminal RNA recognition sites followed by a C-terminal glycine rich region [13]. This family of proteins shuttle between nucleus and cytoplasm and perform multiple functions in terms of pre-mRNA splicing, nuclear import and cytoplasmic trafficking of mRNA, mRNA stability and turnover, and translation [27]. In brain and neuronal cell lines, hnRNP A3 is found mainly in the nucleus, less so within cytoplasm [19]. In keeping with these experimental findings, in the present study, we observed hnRNP A3 immunostaining mainly in the nucleus, though this was highly variable with nuclei densely staining in some cases but remaining unstained in others. There appeared to be no consistent staining pattern across different disease or control states suggesting that this lack of consistent stainability may represent a technical, rather than physiological, change. However, against this, we noted a consistent moderate to strong nuclear staining for hnRNP A1 and A2/B1, even in those cases where there was no nuclear staining for hnRNP A3. Hence, it is still not clear whether this lack of staining represents a physiological/pathological clearance of nuclear protein or whether it reflects variable preservation of antigen in post mortem brain.

Because Mori et al. found hnRNP A3 to bind directly to G4C2 repeats [22], and because hnRNP A3 is known to act in RNA export [19], it is possible that an aberrant export of *C9orf72* pre-mRNA, or the non-ATG translation thereof, could be triggered specifically by an increased binding of hnRNP A3. This might explain why this member of hnRNP A/B family is associated with DPR, whereas hnRNP A1 and A2/B1 are not associated with DPR even though they are able to bind to the G4C2 sequence in pull down assays [22]. However, if this is so, it remains unclear why only a proportion of p62-immunoreactive DPR apparently contain hnRNP A3 protein, at least as can be detected immunohistochemically, and why the proportion of cells and cell types displaying hnRNP A3 immunostaining in cases with *C9orf72* expansions varies so widely. Mori et al. [23] also noted that reduced hnRNPA3 expression in *C9orf72* cases leads to increased levels of the repeat RNA as well as enhanced production and deposition of DPR proteins and RNA foci. Hence, reductions in physiological levels of hnRNP A3, consequent to binding to DPR, might also facilitate production of RNA foci, as well as DPR formation.

The exact (chemical) nature of the binding between hnRNP A3 and DPR is unclear. DPRs (and RNA foci) cause dysfunction of nucleocytoplasmic transport and result in hnRNP A3 mislocalization. HnRNP A3 may then gather in stress granules and combine with arginine rich poly GR/PR DPR species through interaction at its low complexity sequence [17, 18]. Nonetheless, this does not account for hnRNP A3-specific colocalization with DPR aggregates since hnRNPA1 and A2/B1 are also partners in the poly GR/PR interactome [17, 18]. However, the glycine-rich low complexity domain of hnRNP A3 is much longer than that of hnRNP A1 and hnRNP A2/B1, and might thus make hnRNP A3 more prone to aggregate. Once trapped by DPR in stress granules, it is then p62-labeled for degradation via the proteasome.

## Conclusions

In summary, in the present study, we have shown that at least a proportion of TDP-43-negative, p62-immunoreactive DPR contain hnRNP A3 protein in cases with *C9orf72* expansions. No other members of hnRNP A/B family were seen to be present in such inclusions. Present data therefore suggest a specific relationship between hnRNP A3 and DPR in inclusion body formation, rather than a simple passive recruitment of hnRNP A3 protein into the aggregating protein conglomerate. The lack of hnRNP A1 and A2/B1 within DPR would be in keeping with such an ‘active recruitment’ of hnRNP A3 into DPR, and the lack of these former hnRNPs in TDP-43 aggregates, while being associated with FUS aggregates (for hnRNP A1 at least), points to important and specific roles for the different hnRNPs in the pathogenesis of the different forms of FTLD.

## Additional file

Additional file 1: Selected clinical, pathological and genetic details on cases studied. (XLSX 17 kb)
